# Identification of biallelic mutations in *MCM3AP* and comprehensive literature analysis

**DOI:** 10.3389/fgene.2024.1405644

**Published:** 2024-08-20

**Authors:** Chan Liu, Qingfeng Xie, Quan Hu, Bingwu Xiang, Kaiyi Zhao, Xiang Chen, Feixia Zheng

**Affiliations:** ^1^ Department of Physical Medicine and Rehabilitation Center, The Second Affiliated Hospital and Yuying Children’s Hospital of Wenzhou Medical University, Wenzhou, Zhejiang, China; ^2^ Department of Pediatrics Neurology, The Second Affiliated Hospital and Yuying Children’s Hospital of Wenzhou Medical University, Wenzhou, Zhejiang, China

**Keywords:** peripheral neuropathy, intellectual disability, *MCM3AP*, GANP, biallelic mutations

## Abstract

**Background:**

Minichromosome maintenance complex component 3 associated protein (*MCM3AP*) is a gene in which mutations can result in autosomal recessive peripheral neuropathy with or without impaired intellectual development. The *MCM3AP* genotype-phenotype correlation and prognosis remain unclear. The aim of this study was to explore the genotype–phenotype correlations pertaining to *MCM3AP*.

**Methods:**

Whole-exome sequencing (WES) combined with copy number variation sequencing (CNV-seq) were performed on the genomic DNA isolated from a Chinese family, and Sanger sequencing, quantitative PCR and cDNA analyses were performed to examine the mutations. The retrospective study was conducted on 28 individuals with biallelic *MCM3AP* mutation-related diseases, including features such as mutations, motor development impairment, intellectual disability, weakness/atrophy, and cerebral magnetic resonance imaging abnormalities.

**Results:**

Sequencing identified novel compound heterozygous mutations in *MCM3AP*, namely, a paternal variant c.1_5426del (loss of exons 1–25) and a maternal splicing variant c.1858 + 3A>G. Functional studies revealed that the variant c.1858 + 3A>G resulted in the heterozygous deletion of exon 5, thereby affecting splicing functionality. Furthermore, the compound heterozygous mutation may affect the functionality of the protein domain. Retrospective analysis revealed different genotype–phenotype correlations for the pathogenic variants in biallelic *MCM3AP*: all individuals (100%) with mutations outside the Sac3 domain exhibited early-onset symptoms, motor developmental delays, and cognitive abnormalities, conversely, the proportions of individuals carrying mutations within the domain were 26.7% (motor delays) and 46.7% (cognitive abnormalities).

**Conclusion:**

Our findings further expand the genetic mutation spectrum of biallelic *MCM3AP* and highlight the genotype-phenotype associations. Additionally, we elaborate on the importance of rehabilitation intervention.

## 1 Introduction

Biallelic mutations in minichromosome maintenance complex component 3 associated protein (*MCM3AP*, MIM*603294) can result in autosomal recessive peripheral neuropathy with or without impaired intellectual development (PNRIID, MIM#618124). This early-onset neurologic syndrome is often progressive and results in distal motor impairment and gait difficulties, frequently accompanied by loss of ambulation and difficulties with manual dexterity. Furthermore, most of the affected individuals present with intellectual disability (ID) or learning difficulties (Schuurs-Hoeijmakers et al., 2013; Mert et al., 2017; Ylikallio et al., 2017; Kennerson et al., 2018; Woldegebriel et al., 2020). Eye movement abnormalities, characteristic hand and foot deformities, and scoliosis are some additional features.

The minichromosome maintenance (MCM) family is highly conserved in vertebrates and comprises *MCM2–MCM10*. These proteins play essential roles in DNA replication and cell cycle progression. *MCM3AP* interacts with the *MCM3* protein, which is involved in the initiation of DNA replication (Abe et al., 2000; Masai et al., 2005). Germinal center-associated nuclear protein (GANP) is a 218 kDa multidomain protein encoded by 28 exons of *MCM3AP* in chromosome 21q22.3; it is ubiquitously expressed and predominantly localized in the nucleus (Abe et al., 2000; Wickramasinghe et al., 2010). It contains an N-terminal domain comprising phenylalanine-glycine repeats, a C-terminal acetyltransferase domain, DNA primase region, suppressor of actin 3 (Sac3) mRNA-binding domain, and the Cdc31-interaction domain (CID) in the middle (Takei et al., 2001; Takei et al., 2002; Ylikallio et al., 2017). However, the overall function of mammalian GANP has not been determined. Functionally, GANP serves as an mRNA export factor; its depletion leads to mRNA accumulation in the nucleus and subsequent cellular degeneration, which is associated with various neurodegenerative disorders (Wickramasinghe et al., 2010; Mert et al., 2017; Ylikallio et al., 2017; Nussbacher et al., 2019). In flies, GANP can suppress TDP-43-mediated degeneration of motor neurons (Sreedharan et al., 2015). Overall, recessive mutations in *MCM3AP* are associated with neuropathy and ID as well as functional impairment of the GANP protein (Ylikallio et al., 2017; Woldegebriel et al., 2020).

Biallelic *MCM3AP*-associated disease has been reported previously; however, the genotype-phenotype correlation pertaining to *MCM3AP* remains unclear, and the prognosis has not been investigated. In this study, we identified a novel compound heterozygous variant of *MCM3AP* and investigated the efficacy of rehabilitation in a Chinese family. Furthermore, we reviewed several previous studies to explore the genetic spectrum of *MCM3AP* and its relationship with the PNRIID phenotype.

## 2 Methods

### 2.1 Clinical evaluation

In this study, patients were recruited from the Department of Rehabilitation, the Second Affiliated Hospital of Wenzhou Medical University. The signs and symptoms of the disease in the proband and his family members were documented. The Ethics Committee of the Second Affiliated Hospital of Wenzhou Medical University approved this study (2021-K-127-02). All procedures were conducted according to the principles outlined in the Declaration of Helsinki (World Medical Association, 2013). Legal guardians provided written informed consent.

### 2.2 Whole-exome sequencing (WES) and copy number variant sequencing (CNV-seq)

Trio-(WES&CNV-seq) were performed to identify the gene mutations in the proband and his parents, and the proband's sister verified the mutations. Ethylenediaminetetraacetic acid (EDTA)-anticoagulated blood was collected, followed by genomic DNA (gDNA) extraction using a blood column medium extraction kit (Kangweishiji, China). The Qubit 2.0 fluorometer and 1.0% agarose gel electrophoresis were utilized to measure DNA quality and concentration, respectively. Subsequently, the gDNA was fragmented, end-repaired, ligated, and subjected to polymerase chain reaction (PCR) to construct a sequencing library. Then, IDT xGen^®^ Exome Research Panel v2.0 was used to hybridize the resulting target DNA fragments for enrichment to generate a whole exome library. The BGI DNBSEQ-T7 platform was used to perform sequencing. Burrows–Wheeler Aligner (BWA) was used to align the sequencing data against the Ensemble reference genome GRCh37/hg19. Thereafter, single nucleotide polymorphisms and indels were analyzed using GATK. Structural variations (SVs) and copy number variations (CNVs) were annotated and ranked by AnnotSV. Chigene (www.chigene.org), an independently developed mutation annotation software, was used to annotate these high-quality mutations and analyze their potential hazards.

### 2.3 Quantitative PCR (qPCR)

Blood samples were anticoagulated with EDTA and subjected to DNA extraction using the magnetic bead method (TIANGEN, China). Specific primers were designed based on the target region. The system was configured using the SYBR qPCR kit (Sigma-Aldrich, United States). qPCR experiments were performed using a two-step method, and the 2^−△△Ct^ method was utilized (Table 1).

**TABLE 1:** qPCR experimental procedure.

Primer	Sequence	Fragment size	Temperature of annealing
ALB-QF	5’-AGT​GCA​CTT​GTT​GAG​CTC​GTG-3’	128	60
ALB-QR	5’-GCA​AAG​CAG​GTC​TCC​TTA​TCG-3’		
MCM3AP-1QF	5’-CAC​CTG​GAA​TCC​GGG​AAG​GTG​C-3’	121	65
MCM3AP-1QR	5’-GGT​CGT​CTG​GGC​AAC​AAG​GAG​G-3’		
MCM3AP-13QF	5’-GCA​GCT​CCC​CGC​AGC​ACG​TA-3’	101	65
MCM3AP-13QR	5’-ACT​GTG​AGG​AAG​TTG​GCT​CTG​CG-3’		
MCM3AP-25QF	5’-GAA​ACA​TAC​TGA​ACA​TGA​ACC​TCA​CCG-3’	100	60
MCM3AP-25QR	5’-AAA​ATA​TGA​TGT​TCC​TTT​GTC​GTG​G-3’		

### 2.4 cDNA analysis

Blood samples were collected from the proband and anticoagulated with EDTA. Then, RNA was extracted using the Blood RNA Extraction Kit (BioTek, China), followed by reverse transcription with random hexamers (Vazyme, China) to obtain cDNA. Based on *MCM3AP* (NM_003906.5 → NP_003897.2), c.1858 + 3(IVS5)A>G-specific primers were designed (Table 2). PCR was performed to amplify the target sequences, followed by 1.0% agarose gel electrophoresis and Sanger sequencing using the ABI3730 sequencer for verification. The results were analyzed using Chromas software.

**TABLE 2:** Primer sequences used for cDNA sequencing.

Variant		Location	Product length
c.1858 + 3(IVS5)A>G	F: 5’- ACA​GAA​GCG​AGA​GCA​CAG​AC -3’	Exon2	994
	R: 5’- TCA​ATC​AGG​GAC​ACC​GTC​AG -3’	Exon8	
References (WDR45: NM_001029896.2 → NP_001025067.1)	F: 5’-CTC​GTC​TGC​TCC​ATT​CAC​GA3’	Exon8	508
	R: 5’-CAC​GTC​GAA​AGC​CTC​TCT​GT3’	Exon11	

### 2.5 Clinical data collection and literature review

Databases such as PubMed, the Web of Science, and Google Scholar were searched using the term “MCM3AP” from inception until February 2024 without language restrictions to retrieve relevant human mutation cases. The clinical and genetic characteristics of the patients were collected.

## 3 Results

### 3.1 Clinical manifestations

**FIGURE 1:**
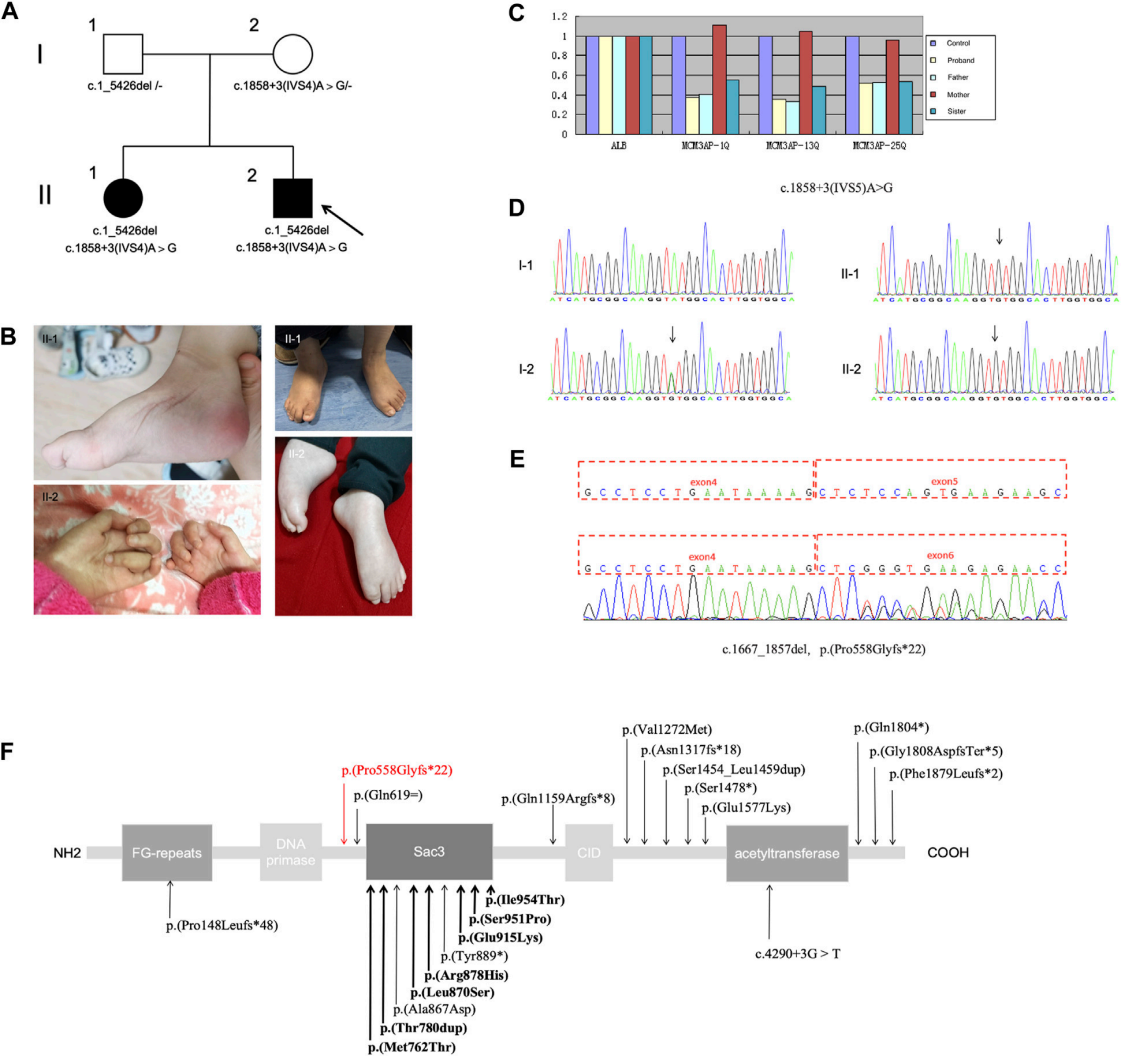
**(A)** Pedigree of the family; the arrow indicates the proband. **(B)** Distal muscle atrophy and contractures accompanied by pes cavus and claw hand. **(C–E)** Presence of the compound heterozygous variants c.1_5426del (loss of exons 1–25) and c.1858 + 3A>G in *MCM3AP.* **(C)** Copy number analysis of exons 1–25 of *MCM3AP* in the affected siblings and parents. **(D)** Chromatograms of the c.1858 + 3A>G variant. **(E)** Functional experiments showing that the splice variant c.1858 + 3A>G (p.Pro558Glyfs*22) results in the deletion of exon 5. **(F)** Schematic illustrating the structure of the GANP protein along with the *MCM3AP* variants; the novel variant is indicated using a red arrow, whereas previously reported variants are indicated in black (bold indicates homozygous variants).

This study outlines the cases of two affected siblings aged 14 and 5 years (sister, II-1; brother, II-2) who were born to nonconsanguineous parents (Figure 1A). They had experienced delayed motor milestones during infancy and had a history of global developmental delay. Patient II-2 presented with mouth twitching and lip cyanosis from the first day of birth, raising the suspicion of convulsions. Patient II-1 could independently sit at 8–9 months of age but was unable to independently stand at 3 years of age. In contrast, patient II-2 was able to independently sit at 14 months of age and independently stand at 3 years of age. Patient II-2 underwent surgery for a right foot deformity at the age of 4 years; however, abnormal posture remained despite independent walking ability. Moreover, both siblings exhibited impaired fine motor skills. After starting rehabilitation at the age of 7 months, patient II-2 exhibited improvements in overall movement. At present, he can walk independently, climb stairs with orthopedic shoes, perform complete gripping and other actions (except for slight atrophy of the thenar muscles and poor thumb movement), and has better cognition than patient II-1. In patient II-1, rehabilitation was initiated at 3.5 years of age; she achieved maximum motor status at 4 years of age and was able to independently walk with aid for short distances. However, she exhibited inflexibility in grasping objects and could not climb stairs. Moreover, upon treatment cessation, her mobility significantly regressed, resulting in an inability to sit independently, stand, and grasp objects. These symptoms were accompanied by weakness and muscle atrophy, which primarily affected the distal region of the limbs, with hand and foot deformities and scoliosis. Patient II-1 also continued to exhibit issues with urinary incontinence. The right lower limb of patient II-2 was thinner than the left lower limb. Both siblings exhibited ID and dysarthria but no paresthesia, hearing impairment, or eye movement disorders. In both cases, right pes cavus and left crural valgus were observed (Figure 1B). Tendon reflexes were absent. Electromyography revealed motor axonal neuropathy, whereas cerebral magnetic resonance imaging (MRI) revealed abnormal signals in the bilateral lateral ventricles. Lastly, electroencephalography revealed epileptic wave discharges in both individuals; however, patient II-1 did not experience seizure attacks.

### 3.2 Genetic findings

Through diagnostic WES combined with CNV-seq, the compound heterozygous mutation c.1_5426del (loss of exons 1–25) and splicing variant c.1858 + 3A>G in *MCM3AP* were identified. Sanger sequencing validated the findings. The mutations observed in *MCM3AP* of the patients were inherited from their parents (both heterozygous; Figure 1A). The identified variants were not observed in databases such as 1,000 Genomes, Exome Aggregation Consortium, dbSNP 150, or the Genome Aggregation Database. The phenotype of the patients was consistent with that of biallelic *MCM3AP*-associated diseases.

Next, qPCR was performed to analyze the copy numbers of exons 1–25 in the target gene *MCM3AP* (Figure 1C). Compared with normal controls, the copy number ratio of exons 1–25 in *MCM3AP* was approximately 0.5 in both siblings and their father, indicating a loss of heterozygosity for exons 1–25. In contrast, the ratio was approximately 1 in the mother, suggesting a normal copy number. The paternally inherited variant potentially resulted in the loss of gene function and was not detected in the normal control population database. Therefore, according to the American College of Medical Genetics and Genomics (ACMG) guidelines (Richards et al., 2015), this variant was classified as a likely pathogenic variant (PVS1 + PM2).

Splicing functional experiments revealed that the maternal variant c.1858 + 3A>G (p.Pro558Glyfs*22) in *MCM3AP* can affect splicing, leading to a transcript without exon 5, which, in turn, results in the domain deletion of Sac3, CID, and acetyltransferase (Figures 1D–F). Subsequently, based on the ACMG standards and guidelines, this variant was classified as PS3 + PM2 + PP3.

Co-segregation studies confirmed that both parents were carriers of *MCM3AP* mutations without any evidence of neurologic symptoms or signs. These findings suggest an association between *MCM3AP* variations and the phenotypic manifestations observed in the affected individuals.

### 3.3 Clinical and genetic characteristics of the patients carrying biallelic mutations in *MCM3AP*

Eight studies reported 26 cases of biallelic *MCM3AP*-associated diseases in 15 families. Two families (patients #19, #20, #21, and #26) were affected by multiple sclerosis (MS). Including our variants, 23 homozygous or compound heterozygous mutations have been identified. Table 3 summarizes the biallelic *MCM3AP* mutations and associated clinical characteristics of the 28 affected individuals.

**TABLE 3:** Clinical characteristics of the patients included in this study and previous studies.

Patient	Age at assessment /Sex	Variants	Sac3 domain	Delayed motor	Walking at age	Loss of ambulation	Cognitive function	Weakness /Atrophy	Tendon reflexes	NCS	MRI findings	Others
1 Schuurs-Hoeijmakers et al. (2013)	NA/M	c.2743G>A p. (Glu915Lys) Homozygous	+	NA	NA	NA	Borderline-mild ID	NA	NA	NA	NA	Hypotonia, ptosis, saccadic eye movement, ataxia, facial dysmorphisms
2 Schuurs-Hoeijmakers et al. (2013)	NA/F		+	NA	NA	NA	Borderline-mild ID	NA	NA	NA	NA	Hypotonia, ptosis, saccadic eye movement, ataxia, facial dysmorphisms
3 Ylikallio et al., (2017)	19 years /M	① c.3814G>A p. (Val1272Met) ② c.443delC p. (Pro148Leufs*48)	—	+	15 months	10–11 years	Learning difficulty, no ID	Upper and lower distal extremity muscles	Accelerated	Sensorimotor axonal neuropathy	Mildl increased signal intensity in the right temporal lobe	Hyperesthesia, visuomotor difficulty, ventilator dependent from age 14, scoliosis, crural valgus, tremor, short stature
4 Ylikallio et al. (2017)	13 years /F		—	+	23 months	10 years	Learning difficulty, no ID	Distal and facial muscle	Normal	Sensorimotor axonal neuropathy	Mild unspecific signal intensity in temporal lobes	Hyperesthesia, hypotonia, intermittent strabismus, finger contractures, pes cavus, urinary incontinence, short stature
5 Ylikallio et al. (2017)	8 years /M		—	+	2 years	Still ambulant (broad-base)	Mild ID	Distal muscle	Absent	Sensorimotor axonal neuropathy	NA	Left amblyopia, microtia, sensorineural hearing impairment, crural valgus, short stature
6 Ylikallio et al. (2017)	8 years /F	① c.2600C>A p. (Ala867Asp) ② c.2667C>A p. (Tyr889*)	+	+	4 years	Still ambulant for short distances	Likely ID	All limbs and velopharyngeal weakness	Absent	Sensorimotor axonal neuropathy	Brain: Normal Spinal: Cervical myelomalacia	Hypoesthesia, bulbar dysfunction, kyphosis, hypermobility, finger contractures, pes planus, dysarthria, swallowing problems, obstructive sleep apnoea, ataxia, tremor, syndactyly
7 Ylikallio et al. (2017)	3 years /F	① c.1857A>G p. (Gln619 = ) ② c.3949_3950insG p. (Asn1317fs*18)	—	+	Not ambulant	Not ambulant	Impaired (BSID-II NL)	All limbs and facial weakness	Absent	Sensorimotor axonal and demyelinaitng neuropathy	NA	Decreased pain sensation, hypotonia, hypermobility, congenital ptosis, esotropia, scoliosis
8 Ylikallio et al. (2017)	28 years /F	① c.4433C>A p. (Ser1478 ② c.4729G>A p. (Glu1577Lys)	—	+	17 months	15 years	Mild ID	Upper and lower distal extremity muscles	Absent	Sensorimotor axonal neuropathy	Normal	Strabismus, foot and hand contractures, obesity, primary ovarian failure
9 Ylikallio et al. (2017)	27 years /F		—	+	20 months	24 years	Mild ID	Upper and lower distal extremity muscles	Absent	Sensorimotor axonal neuropathy	Mild ventriculomegaly, white matter cysts in the left posterotemporal parietal region	Strabismus, corneal hydrops, velopharyngeal insufficiency, dysarthria, foot and hand contractures, seizures, depression, obesity, primary ovarian failure
10 Ylikallio et al. (2017)	20 years /M	c.2285T>C p. (Met762Thr) Homozygous	+	+	4 years (with aid)	10 years	Impaired (IQ not available)	Distal and proximal muscles	Absent	Sensorimotor neuropathy	NA	Distal hypoesthesia, ataxia
11 Ylikallio et al. (2017)	12 years /M		+	—	1 year	NA	Impaired (IQ not available)	All limbs (distal than proximal)	Absent	Sensorimotor demyelinating neuropathy	NA	Distal hypoesthesia
12 Mert et al., (2017)	8 years /F	c.2633G>A p. (Arg878His) Homozygous	+	+	4 years	Ambulant (gait problems)	Mild ID	Upper and lower distal extremity muscles	Absent	Sensorimotor axonal neuropathy	Normal	Strabismus, ophthalmoparesis, scoliosis
13 Mert et al., (2017)	30 years /F	c.2851T>C p. (Ser951Pro) Homozygous	+	—	Normal	Ambulant (gait problems)	Normal	Upper and lower distal extremity and distal spinal muscles	Absent	Motor neuropathy	Normal	Stuttering, pes cavus, claw hands
14 Mert et al. (2017)	27 years /F		+	—	Normal	Ambulant (gait problems)	Normal	Upper and lower distal extremity and distal spinal muscles	Absent	Motor axonal neuropathy	NA	Pes cavus, claw hands
15 Mert et al. (2017)	13 years /F	① c.5410C>T p. (Gln1804*) ② c.4360_4377dup18 p. (Ser1454_Leu1459dup)	—	+	3 years	Ambulant (gait problems)	Mild ID	Upper and lower distal extremity muscles	Absent	Sensorimotor axonal neuropathy	Minimal colpocephaly	Intermittent strabismus, myopic astigmatism, conductive hearing loss, kyphosis, pes cavus
16 Kennerson et al. (2018)	23 years /F	c.2609T>C p. (Leu870Ser) Homozygous	+	—	Normal	Ambulant but unsteady	Normal	Upper and lower distal extremity muscles	Normal	Sensorimotor axonal neuropathy	Middle fossa arachnoid cysts	Hypoesthesia, obstructive sleep apnoea, pyschosis, obesity, diabetes type 2
17 Kennerson et al. (2018)	25 years /M		+	—	Normal	Ambulant but unsteady	Normal	Upper and lower distal extremity muscles	Normal	NA	Normal	Hypoesthesia, obstructive sleep apnoea, obesity
18 Kennerson et al. (2018)	21 years /F		+	—	Normal	Ambulant but unsteady	Normal	Upper and lower distal extremity muscles	Normal	Motor axonal neuropathy	NA	Hypoesthesia, obstructive sleep apnoea, obesity, diabetes type 2, episgastric hernia repair
19 Sedghi et al. (2019)	30 years /F	c.2861T>C p. (Ile954Thr) Homozygous	+	—	12 months	23 years	Normal	Upper and lower distal extremity muscles	Absent	Motor axonal neuropathy	Brain: Multiple plaque in peri and para ventricular, juxta cortical, infra tentorial and corpus callous with enhancing plaque Spinal: Cord atrophy	MS, paresthesias (in back), distal hypoesthesia, hypotonia, right hemiparesis, diplopia, vertigo, hammertoes, finger and Achilles contractures, tremor, urinary incontinence
20 Sedghi et al. (2019)	39 years /M		+	—	14 months	39 years	Normal	Upper and lower distal extremity muscles	Absent	Motor axonal neuropathy	Brain: Multiple plaque in peri and para ventricular, juxta cortical, infra tentorial and corpus callous with one enhancing lesion and brain atrophy Spinal: Cord atrophy	MS, hypotonia, diplopia, pseudobulbar affect, dysarthria, hammertoes, seizure, tremor, urinary incontinence
21 Sedghi et al. (2019)	44 years /F		+	—	13 months	Still ambulant	Normal	Upper and lower distal extremity muscles	Absent	Motor axonal neuropathy	Multiple plaque in para and peri ventricular, corpus callous, juxta- cortical and nfratentorial (pons) spaces	MS, hypoesthesia, diplopia, pseudobulbar affect, dysarthria, hammertoes, ataxia, tremor, urinary incontinence
22 Woldegebriel et al. (2020)	20 s years /M	① c.3814G>A p. (Val1272Met) ② c.5423delG p. (Gly1808AspfsTer*5)	—	+	12 months (standing)	26–27 years or earlier	Moderate -severe ID	+	NA	NA	NA	Spastic diplegia, dysarthria, generalized epilepsy, ataxia, aggression and behavioral problems
23 Woldegebriel et al. (2020)	20 s years /F		—	+	12 months (standing)	26–27 years or earlier	Moderate -severe ID	+	NA	NA	NA	Spastic diplegia, dysarthria, generalized epilepsy, ataxia, aggression and behavioral problems
24 Woldegebriel et al. (2020)	4 years /F	① c.4290+3G > T ② c.3476_3477del p. (Gln1159Argfs*8)	—	+	Not ambulant	Not ambulant	+	+	NA	Sensorimotor neuropathy	Hyperintensity of basal ganglia and mild ventriculomegaly	Hypotonia, abnormal (dyskinetic) movements. ventilator dependent after recurrent airway infections, swallowing problems, scoliosis
25 Dong et al. (2020)	11 years /F	① c.2633G>A, p. (Arg878His) ② c.5634-1G>T p. (Phe1879Leufs*2	+/−	+	17 months	Still ambulant	Normal	Upper and lower distal extremity muscles	Absent	Sensorimotor axonal neuropathy	NA	Distal numbness, pes cavus
26 Yuksel et al. (2023)	12 years /M	c.2337_2339dup p. (Thr780dup) Homozygous	+	+	Not ambulant	Not ambulant	Moderate cognitive retardation	+	Absent	Sensorimotor demyelinating neuropathy	Multiple hyperintensity in pericallosal -Dawson’s fingers, splenial, juxtacortical, left frontal and infratentorial	MS, hypotonia, facial paralysis and left hemiparesis, ophthalmoparesis, hypertelorism, scoliosis, pes cavus
27 (Present Study)	14 years /F	① c.1858+3A>G p. (Pro558Glyfs*22) ② c.1_5426del loss of exons 1–25)	—	+	4 years (with aid)	4.5 years	Mild - moderate ID	Upper and lower distal extremity muscles	Absent	Motor axonal neuropathy	Hyperintensity in peri and para ventricular space	Ankle hypertonia, dysarthria, scoliosis, claw hands, foot contractures, urinary incontinence, subclinical epilepsy
28 (Present Study)	5 years /M		—	+	4 years	Still ambulant	Borderline ID	Upper and lower distal extremity muscles	Absent	Motor axonal neuropathy	Hyperintensity in peri and para ventricular space	Ankle hypertonia, dysarthria, foot contractures, hammertoes, cryptorchidism, subclinical epilepsy

BSID-II, bayle scales of infant development, Second Edition; ID, intellectual disability; IQ, intelligence quotient; MRI, magnetic resonance imaging; MS, multiple sclerosis; NA, not available; NCS, nerve conduction studies.

Among the 16 families, seven exhibited homozygous mutations (all within the Sac3 domain), and nine exhibited compound heterozygous mutations (seven outside the Sac3 domain, one within the Sac3 domain, and one was a mixed mutation). In the literature cohort, early-onset symptoms accompanied by motor developmental delays were observed in 100.0% (12/12) of individuals carrying mutations outside the Sac3 domain of GANP, and in 26.7% (4/15) of individuals carrying mutations within the Sac3 domain. ID was observed in 17 out of 28 individuals, with two individuals experiencing learning difficulties specifically associated with this condition. Among them, 100% (12/12) of the individuals carrying mutations outside the Sac3 domain presented with cognitive abnormalities, compared to 46.7% (7/15) of those with mutations within the domain.

## 4 Discussion

In this study, we summarized the clinical findings of two patients from a Chinese family with PNRIID who exhibited typical symptoms. The affected individuals presented with slow-progressive distal motor neuropathy, resulting in weakness and atrophy of the foot and/or hand muscles as well as joint deformities, accompanied by ID, dysarthria, urinary incontinence, and cryptorchidism. WES combined with CNV-seq revealed compound heterozygous variants in *MCM3AP*. These variants segregated with the disease phenotype, and the affected individuals carried both variants, whereas their father was a heterozygous carrier of a large fragment deletion, c.1_5426del (loss of exons 1–25), and their mother was heterozygous for c.1858 + 3A>G, a novel splicing variant resulting in the heterozygous deletion of exon 5. Coincidentally, these mutations were passed on to the siblings, eventually leading to the homozygous deletion of exon 5.

GANP, encoded by *MCM3AP*, is a protein associated with neurologic disorders in humans; defects in this protein have been described as causative agents of autosomal recessive Charcot–Marie–Tooth disease with ID (Ylikallio et al., 2017). The disease is characterized by progressive symmetrical distal limb muscle weakness and atrophy, with or without hypoesthesia, and weakened or absent tendon reflexes in the limbs (Mert et al., 2017). The affected individuals primarily exhibited developmental delay from infancy or experienced developmental regression following early normal motor development. To date, 23 pathogenic variants of *MCM3AP* have been identified in 16 families. The vast majority are missense and frameshift mutations. In our study, CNV-seq combined with WES enabled us to identify a novel variant c.1_5426del (loss of exons 1–25) in *MCM3AP* gene, a large pathogenic fragment deletion. These results suggest that CNV-seq or multiplex ligation-dependent probe amplification (MLPA) may be optimal for further evaluation in patients with highly suspected genetic motor/ID disorders who remain undiagnosed after gene panel testing or WES. Disease-associated *MCM3AP* variations can be broadly divided into two classes: those occurring within or outside the Sac3 domain of GANP (Woldegebriel et al., 2020). Notably, the Sac3 domain exhibits high homology across vertebrates (Jones et al., 2000; Jani et al., 2009; Kopytova et al., 2010), where it plays a vital role in exporting mRNAs via nuclear pores into the cytoplasm (Wickramasinghe et al., 2010). The effect of Sac3 on actin and microtubule functions may be a pathological mechanism underlying motor neuron degeneration (Bauer and Kölling, 1996). Among the 16 families, most mutations within the Sac3 domain are homozygous. Literature analysis (Mert et al., 2017; Ylikallio et al., 2017; Kennerson et al., 2018; Ylikallio et al., 2018) revealed the genotype–phenotype correlation of the pathogenic variation in *MCM3AP*, with a tendency toward a milder and slower-progressing phenotype in individuals with homozygous mutations within the Sac3 domain. In contrast, compound heterozygous mutations outside the Sac3 domain lead to early-onset and severe disease courses. In our study, we identified a homozygous exon 5 deletion, which was located outside the Sac3 domain; this deletion possibly affected mRNA stability and resulted in early-onset symptoms with rapid progression. Statistical analysis revealed that all the affected individuals carrying mutations outside the Sac3 domain presented early-onset symptoms accompanied by motor developmental delays. Notably, one severely affected individual carrying mutations outside the Sac3 domain experienced loss of independent ambulation during adolescence and required ventilator support (Ylikallio et al., 2017). Furthermore, missense variants within the Sac3 domain did not lead to GANP protein depletion; however, compound heterozygous *MCM3AP* variants outside this domain drastically decreased GANP protein levels in the nuclear envelope (Woldegebriel et al., 2020). GANP protein depletion and/or loss of function impair the transcription and/or export of mRNAs, resulting in the distinct neurologic disease phenotypes associated with *MCM3AP* mutations.

Previous studies have revealed *MCM3AP* expression in the brain and neuronal tissues (Schuurs-Hoeijmakers et al., 2013). In addition to peripheral neuropathy, ID was also observed as another prominent feature, presenting in 60.7% (17/28) patients. Furthermore, mutations outside the Sac3 domain may predispose individuals to ID. Some affected individuals exhibited other central nervous system (CNS) signs, including seizures (6/28, 21.4%), ataxia (7/28, 25.0%), tremors (5/28, 17.9%), and psychopsychic abnormalities (4/28, 14.3%). In the present study, electroencephalography revealed frequent epileptiform activity in both siblings, with the suspicion of convulsions in patient II-2. Woldegebriel et al. (2020) reported the case of a family, carrying mutations outside the Sac3 domain, that displayed a motor phenotype with progressive encephalopathy as an additional feature. According to the literature, cerebral MRI abnormalities have been observed in 70.6% (12/17) of the cases studied to date. Moreover, GANP expression has been detected in the brain (Abe et al., 2000), with suggestions of its involvement in CNS-originating neoplasms (Ohta et al., 2009). Collectively, these findings suggest the emerging role of GANP, not only in peripheral nerves but also within the CNS. Furthermore, the progression of *MCM3AP*-associated disease to peripheral neuropathy and MS with white matter lesions in the CNS has been described in two families (Sedghi et al., 2019; Yuksel et al., 2023). It has been reported that variants in *MCM3AP* encoding GANP can also cause complex phenotypes such as immunodeficiency, genomic instability, and skin changes (Gatz et al., 2016). The pathogenic mechanism of MS is considered to be immunologically mediated, involving demyelination, with B cells contributing to the immune inflammatory response. The role of GANP in B cell antibody maturation has been suggested (Kuwahara et al., 2004; Singh et al., 2013). The pathogenic mechanism of *MCM3AP* variants may involve neuroimmune damage. Further studies in additional families are needed to clarify these associations and explore the potential pathogenesis further.

The pathogenic variation in *MCM3AP* primarily results in motor neuropathy, with a less pronounced effect on sensory function; however, approximately 42.9% (12/28) of the patients revealed sensory disorders clinically, which primarily manifested as decreased or absent sensation of the cuff and sock distribution. Other potentially associated symptoms included eye movement disorders (10/28, 35.7%), dysarthria (8/28, 28.6%), respiratory dysfunction (6/28, 21.4%), obesity (5/28, 17.9%), and ovarian failure (2/28, 7.1%). In addition, cryptorchidism, which may be a novel PNRIID phenotype, was observed in our case. Studies (Ylikallio et al., 2017; Zhou et al., 2023) have shown that *MCM3AP* is involved in the DNA replication process, and this association may result in a phenotype of steroid hormonal dysregulation.

Rehabilitation, orthotics, and supportive care are some of the currently available treatment modalities. Evidence suggests that mild to moderate exercise is effective and safe (Chetlin et al., 2004; Young et al., 2008). In the present study, both patients exhibited improvements in limb strength and walking ability during rehabilitation; however, patient II-1 experienced functional degeneration as well as joint contracture upon rehabilitation cessation. As the disease progresses, most patients develop joint deformities, with foot and/or hand deformities (16/28, 57.1%) and scoliosis (8/28, 28.6%) being observed in the affected individuals. Surgery can be performed to treat skeletal deformities, including foot or hand deformities or scoliosis, to relieve pain and improve function (Karol and Elerson, 2007). Furthermore, orthotic and assistive devices can be helpful. Patient II-2 underwent plantar fasciotomy to correct the cavus deformity, which improved gait abnormalities and allowed better navigation of stairs while wearing orthopedic shoes. Our study findings highlight the efficacy of rehabilitation interventions for two siblings exhibiting similar clinical manifestations. Patient II-1 missed the rehabilitation period, resulting in functional regression; in contrast, the function (including cognitive) of patient II-2 continued to improve during convalescence. Due to an insufficient sample size, we cannot be certain about the efficacy of rehabilitation; therefore, an increase in the number of reported cases will help clarify this. In addition, PNRIID represents a type of progressive neuropathy. Ongoing research on upstream biological disorders and neurotrophic factors holds promise for mitigating neurodegeneration and instilling optimism among patients.

## 5 Conclusion

We presented the findings of a Chinese family with PNRIID harboring a novel compound heterozygous mutation in *MCM3AP*. We identified a large fragment deletion in *MCM3AP* and confirmed that the splicing variant c.1858 + 3A>G causes the deletion of exon 5. Furthermore, the genotype–phenotype correlation analysis of reported cases of biallelic *MCM3AP*-associated disease suggests that compound heterozygous mutations outside the Sac3 domain may lead to ID and more severe peripheral nerve phenotypes compared to homozygous mutations within the Sac3 domain. The molecular sub-regional location of variants and genotypes helps explain the phenotypic heterogeneity of patients with *MCM3AP* variants. More cases will help clarify genotype–phenotype correlations. Finally, our findings highlight the importance of rehabilitation interventions.

## Data Availability

The data presented in the study are deposited in the Genome Variantion Map repository, accession number GVM000827.
